# *Staphylococcus aureus* Infections in US Veterans, Maryland, USA, 1999–20081

**DOI:** 10.3201/eid1703.100502

**Published:** 2011-03

**Authors:** LaRee A. Tracy, Jon P. Furuno, Anthony D. Harris, Mary Singer, Patricia Langenberg, Mary-Claire Roghmann

**Affiliations:** Author affiliations: University of Maryland, Baltimore, Maryland, USA (L.A. Tracy, J.P. Furuno, A.D. Harris, P. Langenberg, M.-C. Roghmann);; Veterans Administration Maryland Health Care System, Baltimore (M.-C. Roghmann);; US Food and Drug Administration, Silver Spring, Maryland, USA (M. Singer)

**Keywords:** Staphylococcus aureus, methicillin resistance, community-acquired, bacteria, infections, CME, staphylococci, skin infections, veterans, research, *Suggested citation for this article:* Tracy LA, Furuno JP, Harris AD, Singer M, Langenberg P, Roghmann M-C. *Staphylococcus aureus* infections in US veterans, Maryland, USA, 1999–2008. Emerg Infect Dis [serial on the Internet]. 2011 Mar [*date cited*]. http://dx.doi.org/10.3201/eid1703.100502

## MEDSCAPE CME

Medscape, LLC is pleased to provide online continuing medical education (CME) for this journal article, allowing clinicians the opportunity to earn CME credit.

This activity has been planned and implemented in accordance with the Essential Areas and policies of the Accreditation Council for Continuing Medical Education through the joint sponsorship of Medscape, LLC and Emerging Infectious Diseases. Medscape, LLC is accredited by the ACCME to provide continuing medical education for physicians.

Medscape, LLC designates this Journal-based CME activity for a maximum of 1 *AMA PRA Category 1 Credit(s)*^TM^. Physicians should claim only the credit commensurate with the extent of their participation in the activity.

All other clinicians completing this activity will be issued a certificate of participation. To participate in this journal CME activity: (1) review the learning objectives and author disclosures; (2) study the education content; (3) take the post-test and/or complete the evaluation at www.medscape.org/journal/eid; (4) view/print certificate.

**Release date: February 25, 2011; Expiration date: February 25, 2012**

## Learning Objectives

Upon completion of this activity, participants will be able to:

- Describe the change in overall incidence of *S. aureus* infections between fiscal years 1999 and 2008 based on a retrospective cohort study using patient-level data in the Veterans Affairs Maryland Healthcare System
- Describe trends in invasive vs noninvasive *S. aureus* infections, changes in methicillin susceptibility, and changes in location of onset and infection site between fiscal years 1999 and 2008 based on the aforementioned study
- Describe hospital infection-control practices that may contribute to declining incidence of invasive *S. aureus* infections

## MEDSCAPE CME Editor

**Karen L. Foster, MA, **Technical Writer-Editor, *Emerging Infectious Diseases. Karen L. Foster, MA, has disclosed no relevant financial relationships*.

## MEDSCAPE CME AUTHOR

**Laurie Barclay, MD, **freelance writer and reviewer, Medscape, LLC. *Disclosure: Laurie Barclay, MD, has disclosed no relevant financial relationships.*

## AUTHORS

*Disclosures:* ***LaRee A. Tracy, MA, PhD; Jon P. Furuno, PhD; Mary Singer, PhD, MD; Patricia Langenberg, PhD;*** *and* ***Mary-Claire Roghmann, MD, MS,*** *have disclosed no relevant financial relationships.* ***Anthony D. Harris, MD, MPH,*** *has disclosed the following relevant financial relationship: served as an advisor or consultant for Ansell on a retrospective database project.*
