# Supplementary material for: Staphylococcus aureus Infections in US Veterans, Maryland, USA, 1999–20081
Source: Emerg Infect Dis. 2011 Mar;17(3):441–8. doi: 10.3201/eid1703.100502 (PMC3165993; doi:10.3201/eid1703.100502)
Supplement: Related texts

# Research article: *Staphylococcus aureus* Infections in US Veterans, Maryland, USA, 1999–2008^1^

**DOI:** 10.3201/eid1703.100502

**Authors:** LaRee A. Tracy, Jon P. Furuno, Anthony D. Harris, Mary Singer, Patricia Langenberg, Mary-Claire Roghmann

**Affiliations:** Author affiliations: University of Maryland, Baltimore, Maryland, USA (L.A. Tracy, J.P. Furuno, A.D. Harris, P. Langenberg, M.-C. Roghmann);; Veterans Administration Maryland Health Care System, Baltimore (M.-C. Roghmann);; US Food and Drug Administration, Silver Spring, Maryland, USA (M. Singer)

**Keywords:** *Suggested citation for this article:* Tracy LA, Furuno JP, Harris AD, Singer M, Langenberg P, Roghmann M-C. *Staphylococcus aureus* infections in US veterans, Maryland, USA, 1999–2008. Emerg Infect Dis [serial on the Internet]. 2011 Mar [*date cited*]. http://dx.doi.org/10.3201/eid1703.100502

## Abstract

Trends in *Staphylococcus aureus* infections are not well described. To calculate incidence in overall *S. aureus* infection and invasive and noninvasive infections according to methicillin susceptibility and location, we conducted a 10-year population-based retrospective cohort study (1999–2008) using patient-level data in the Veterans Affairs Maryland Health Care System. We found 3,674 *S. aureus* infections: 2,816 (77%) were noninvasive; 2,256 (61%) were methicillin-resistant *S. aureus* (MRSA); 2,517 (69%) were community onset, and 1,157 (31%) were hospital onset. Sixty-one percent of noninvasive infections were skin and soft tissue infections; 1,112 (65%) of these were MRSA. Ten-year averaged incidence per 100,000 veterans was 749 (± 132 SD, range 549–954) overall, 178 (± 41 SD, range 114–259) invasive, and 571 (± 152 SD, range 364–801) noninvasive *S. aureus* infections. Incidence of all *S. aureus* infections significantly increased (p<0.001), driven by noninvasive, MRSA, and community-onset infections (p<0.001); incidence of invasive *S. aureus* infection significantly decreased (p<0.001).

*Staphylococcus aureus* exists as a commensal organism living on the human body in equilibrium with other bacteria and as a common agent associated with a spectrum of diseases ranging from mild, noninvasive skin and soft tissue infections (SSTIs) to invasive, life-threatening bloodstream infections. Increasing incidence of infections caused by methicillin-resistant *S. aureus* (MRSA) has complicated treatment of *S. aureus* infection. Previously MRSA infections were problematic primarily among hospitalized persons or persons exposed to the health care settings. However, since the 1990s, MRSA infections have become more prevalent in healthy, younger persons who have little to no exposure to health care settings. Of particular concern is the rapid increase in MRSA SSTIs reportedly driven by emergence of a new MRSA strain, USA300 (*1**,**2*).

Despite these changes, the epidemiology of *S. aureus* infection, particularly the total effect of infection in the United States, is not well described. Several population-based studies on *S. aureus* infections exist; however, these studies focused on hospital-based populations (*3**–**6*), MRSA infection (*7**–**9*), non-US populations (*10**–**12*), or only estimated the impact of invasive *S. aureus* disease (*10**,**13**–**15*). Additionally, population-level changes in incidence, particularly before and after USA300 MRSA emerged, are largely unknown. To describe overall trends and recent changes in the incidence of *S. aureus* infection while differentiating between invasive and noninvasive, community- and hospital-onset, and methicillin-susceptible and -resistant *S. aureus* infections, we conducted a retrospective population-based study.

## Methods

### Data Source

Our study used data from the Veterans Affairs Maryland Health Care System (VAMHCS*)* over a 10-year period (1999–2008). VAMHCS, a large, integrated health care system, comprises 3 medical centers (Baltimore VA Medical Center, Perry Point VA Medical Center, and the Baltimore VA Rehabilitation and Extended Care Center), ≈730 inpatient beds, and 5 community-based outpatient clinics. VAMHCS uses an electronic health information system known as the Veterans Health Information Systems and Technology Architecture (VistA). This system is used to collect and maintain all health information at each VA medical facility, including the VAMHCS. VAMHCS’s electronic medical records and administrative data, including codes from the International Classification of Diseases, 9th Revision, Clinical Modification (ICD-9-CM), and microbiology culture results collected from the VistA served as the primary data source for this study. The VAMHCS Research and Development Committee and the University of Maryland, Baltimore Institutional Review Board approved this study. Given that data were retrospectively obtained from VistA, informed consent requirements were waived.

### S. aureus Culture Collection and Classification

We collected data during October 1, 1998–September 30, 2008, on all *S. aureus*–positive blood and clinical cultures (excluding surveillance and fecal and nasal cultures) identified through microbiologic accession number, date, time, and specimen type (e.g., blood, skin). Each positive culture was classified as originating from a sterile or a nonsterile body site. We defined a sterile body site according to Centers for Disease Control and Prevention Active Bacterial Core surveillance criteria, and all other body sites were classified as nonsterile (*13*). We defined a unique culture as the first *S. aureus*–positive culture obtained from a patient during a 6-month period. If cultures were obtained from a sterile and a nonsterile site from the patient during the same period, we chose the culture from the sterile site.

For *S. aureus*–positive cultures obtained during an outpatient visit for which the patient was not subsequently hospitalized within a 72-hour period after culture, we obtained all ICD-9-CM codes associated with all of the patient’s outpatient visits on the day of culture. For *S. aureus*–positive cultures obtained during an outpatient visit for which the patient was subsequently hospitalized within a 72-hour period after culture, we collected the outpatient and hospital discharge ICD-9-CM codes. Finally, we obtained all ICD-9-CM discharge codes for *S. aureus*–positive cultures obtained during a hospitalization. Using information from previous studies, we developed a comprehensive list of ICD-9-CM codes for *S. aureus*–related infections and categorized them by the site of infection most consistent with the associated code (*16*).

We determined an invasive *S. aureus* infection on the basis of *S. aureus* isolation from a clinical or blood culture from a normally sterile body site, such as blood, cerebrospinal fluid, pleural fluid, pericardial fluid, peritoneal fluid, joint/synovial fluid, bone, internal body site, and muscle. Identification of noninvasive *S. aureus* infection was based on isolation of *S. aureus* from a clinical culture of a nonsterile site, without a concurrent culture from a sterile site obtained during the same 6-month period, and at least 1 ICD-9-CM code for *S. aureus*–related infection from the outpatient visit or hospitalization associated with the positive culture. For a noninvasive infection, we based the requirement of a matching ICD-9-CM code along with positive culture on concerns that *S. aureus* obtained from a sample from a nonsterile body site can represent either infection or colonization. In a substudy that used a random sample of cases, we estimated that an ICD-9-CM code for *S. aureus*–related infection plus positive clinical culture (from a nonsterile site) increases the probability of a true noninvasive *S. aureus* infection by ≈23.8% over positive clinical culture alone (*16*).

Positive cultures obtained after the first 48 hours of hospitalization, rehabilitation, or long-term stay were classified as hospital onset and all others as community onset. All *S. aureus* infections were classified according to methicillin susceptibility on the basis of in vitro susceptibility to oxacillin. All MRSA infections were grouped into the following epidemiologic categories: health care–associated community onset, defined as cases in persons with at least 1 listed risk factor in the past 12 months; health care–associated hospital onset, defined as cases in persons who have a positive culture within 48 hours after hospitalization; or community-associated, defined as cases in persons with no documented health care–associated community-onset risk factor (*13*). To determine a risk factor for health care–associated community-onset infection, we obtained history of hospitalization, surgery, residence in a long-term care facility, or prior MRSA-positive culture in the past 12 months before the date and time of each index positive MRSA culture. Site of infection (bone or joint, skin or soft tissue, endovascular, respiratory, intraabdominal/pelvic, central nervous system, urinary tract, *S. aureus*–nonspecific site, bacteremia without focus, and other or site not specified) was determined for each *S. aureus* infection on the basis of matching ICD-9-CM code. When cultures matched with multiple ICD-9-CM codes, we chose the highest ranking site of infection on the basis of the likelihood that a culture represented true *S. aureus* infection (*16*).

### Statistical Analysis

Information about annual number of unique veterans, admissions, and total inpatient days for hospitalization, long-term care, and residential rehabilitation programs were obtained from the VAMHCS Medical Administrative Service fiscal year (FY) databases. Annual and 10-year averaged incidence rates per 100,000 veterans were estimated overall and for community-onset infections and per 100,000 inpatient days for hospital-onset *S. aureus* infections. To account for the first 48 hours in the definition for hospital-onset infection, we adjusted inpatient days (number of annual inpatient days minus 2× the number of annual admissions) (*13*). Because the average inpatient length of stay in the VAMHCS is >48 hours, this adjustment provides a consistent and reasonably accurate estimate of inpatient days past 48 hours.

Trends in all *S. aureus* infections were initially assessed by plotting natural and cubic spline smoothers to the observed data plotted as a function of time. The formal analysis of trends of all *S. aureus* infections was based on generalized linear models, assuming a Poisson distribution with a log link function, including FY as a predictor variable and log total number of unique veterans or log-adjusted inpatient days as an offset variable (*17*). Model fit was assessed by evaluating the deviance and Akaike information criterion, and regression coefficients for trend were assessed by the partial Wald test (*18**,**19*). Additional models were fit for each stratum of interest, i.e., invasive, noninvasive, onset, and methicillin susceptibility. Analyses were performed by using SAS version 9.1 (SAS Institute Inc., Cary, NC, USA) and R version 2.7.0 (2008) software (www.r-project.org).

## Results

For FY 1999–FY 2008, a mean (± SD) of 48,940 (± 3,926) unique veterans accessed care in the VAMHCS each year. The mean annual numbers of acute care and nursing home or intermediate care admissions was 5,854 (± 199) and 919 (± 161) corresponding to 23,183 (± 1,743) and 98,902 (± 10,893) inpatient days, respectively.

### Overall Incidence of S. aureus Infection

We identified 3,674 *S. aureus* infections, of which 2,816 (77%) were noninvasive and 2,256 (61%) were MRSA. The overall proportion of community-onset and hospital-onset infections was 2,517 (69%) and 1,157 (31%), respectively. The 10-year averaged incidence per 100,000 veterans was 749 cases (± 132 SD, range 549–954) overall, 178 (± 41 SD, range 114–259) invasive, and 571 (± 152 SD, range 364–801) for noninvasive *S. aureus* infections. The annual incidence per 100,000 veterans of all *S. aureus* infections increased significantly starting in 2003 (p<0.001). This increase was driven by significant (p<0.001) increases in noninvasive, MRSA, and community-onset infections (Figure 1).

**Figure 1:** Incidence per 100,000 veterans of *Staphylococcus aureus* infections by invasive and noninvasive (A), methicillin susceptibility (B), and onset (C), Veterans Affairs Maryland Health Care System, fiscal years 1999–2008. Solid line represents all *S. aureus* infections. MRSA, methicillin-resistant *S. aureus*; MSSA, methicillin-susceptible *S. aureus*; CO, community onset; HO, hospital onset.

### Invasive *S. aureus* Infections

We identified 858 invasive *S. aureus* infections, of which 75% were based on positive blood cultures, among 800 unique veterans during FY 1999–FY 2008. The proportions of community- and hospital-onset invasive *S. aureus* infections were 56% and 44%, respectively; 52% were caused by MRSA (Table 1). Among all 449 invasive MRSA infections, 243 (54%) were epidemiologically classified as health care–associated hospital-onset, 152 (34%) as health care–associated community-onset, and 54 (12%) as community-associated MRSA.

**Table 1:** Characteristics of *Staphylococcus aureus* infections, Veterans Affairs Maryland Health Care System, fiscal years 1999–2008*

Characteristic	Invasive *S. aureus* infections							Noninvasive *S. aureus* infections					
	Total, n = 858	Onset			Susceptibility			Total, n = 2,816	Onset			Susceptibility†	
		H, n = 381	C, n = 477		MRSA, n = 449	MSSA, n = 409			H, n = 778	C, n = 2,038		MRSA, n = 1,807	MSSA, n = 1,006
No. patients	800	359	441		415	385		2,511	708	1,803		1,600	908
Age, y‡													
Mean (SD)	64 (14)	67 (13)	61 (13)		65 (13)	62 (14)		62 (14)	68 (13)	60 (14)		62 (14)	62 (14)
Median	64	70	60		65	61		61	71	58		61	60
Range	28–98	28–98	30–94		28–97	29–98		19–94	19–93	22–94		23–94	19–93
Male sex, no. (%)	782 (98)	370 (97)	469 (98)		399 (98)	440 (98)		2,436 (97)	691 (98)	1,745 (97)		1,552 (97)	881 (97)
Race, no. (%)													
Black	393 (49)	154 (43)	239 (54)		190 (48)	203 (53)		1,212 (48)	289 (41)	923 (51)		781 (49)	429 (47)
White	387 (48)	200 (56)	187 (42)		217 (52)	170 (44)		1,186 (47)	412 (58)	774 (43)		745 (47)	440 (49)
Other§	20 (3)	5 (1)	15 (3)		8 (2)	12 (3)		113 (5)	7 (1)	106 (6)		74 (5)	39 (4)

*MRSA, methicillin-resistant *S. aureus*. MSSA, methicillin-susceptible *S. aureus*; H, hospital; C, community. †Methicillin-susceptibility unknown for 3 patients with noninvasive *S. aureus* infections. ‡Age unknown for 8 patients with noninvasive *S. aureus* infections. §Includes missing data. Other/unknown race not included in test.

The annual incidence of all invasive *S. aureus* infections decreased gradually from FY 1999 through FY 2008 (Figure 1, panel A). In FY 1999, the estimated incidence of invasive *S. aureus* infection was 259 infections per 100,000 veterans; however, by FY 2008, incidence was 114 per 100,000 veterans (p<0.001). This decrease appears to be associated with overall decreases in the incidence of invasive MRSA (135–68/100,000; p = 0.02) and methicillin-susceptible *S. aureus* (MSSA) (123–47/100,000; p = 0.009).

The incidence of invasive hospital-onset *S. aureus* infections decreased from 150 to 85 cases per 100,000 adjusted acute-care inpatient days (Table 2). Invasive hospital-onset MSSA and MRSA infections significantly decreased during the 10-year period (p = 0.01 and p<0.001, respectively). The incidence of invasive community-onset *S****.*** *aureus* infections decreased 1.9-fold from 119 to 64 per 100,000 veterans and was driven by decreases in incidence of invasive community-onset MSSA. Incidence of invasive community-onset MRSA remained relatively unchanged (46 to 41/100,000 veterans).

**Table 2:** Incidence and type of *Staphylococcus aureus* infections, Veterans Affairs Maryland Health Care System, fiscal years 1999–2008*

Fiscal year	Invasive infection						Noninvasive infection				
	Hospital onset†			Community onset‡			Hospital onset†			Community onset‡	
	MSSA	MRSA		MSSA	MRSA		MSSA	MRSA		MSSA	MRSA
1999	46.3	104.1		72.6	46.0		88.7	250.7		246.7	113.7
2000	58.4	75.1		72.4	44.4		116.8	183.5		142.5	100.5
2001	50.6	109.6		66.3	27.8		139.1	273.9		117.6	119.7
2002	58.1	116.1		57.5	31.7		111.7	254.6		150.8	107.1
2003	53.2	87.1		59.0	43.3		135.5	222.6		104.2	114.0
2004	24.3	72.8		59.2	51.3		140.8	301.0		165.8	286.3
2005	33.5	54.4		47.1	41.2		62.8	272.1		176.6	467.0
2006	59.3	59.3		57.5	46.0		91.2	150.4		164.8	360.2
2007	29.3	37.7		46.3	48.3		87.9	196.8		162.2	451.7
2008	40.3	44.3		23.3	40.7		64.4	221.5		149.2	397.2
Average	45.3	76.0		56.1	42.1		103.9	232.7		158.0	251.8

*MSSA, methicillin-susceptible *S. aureus*; MRSA, methicillin-resistant *S. aureus*. †Incidence per 100,000 adjusted acute-care inpatient days. ‡Incidence per 100,000 veterans.

### Noninvasive S. aureus Infections

We identified 2,816 noninvasive *S. aureus* infections among 2,511 unique patients during FY 1999–FY 2008. Overall, 28% and 72% of *S. aureus* infections were noninvasive hospital onset and community onset, respectively; 1,807 (64%) were caused by MRSA and 1,006 (36%) by MSSA (Table 1). Of MRSA infections, 539 (30%), 572 (32%), and 696 (39%) were epidemiologically classified as health care–associated community-onset, health care–associated community-onset, and community-associated MRSA infections, respectively.

From FY 1999 through FY 2008, the overall incidence of noninvasive *S. aureus* infections increased significantly (p<0.001) (Figure 1, panel A). Incidence in FY 1999 was 576 cases per 100,000 veterans and rapidly increased beginning in FY 2003, peaking at 801 cases per 100,000 veterans by FY 2005. These increases were driven primarily by increases in noninvasive MRSA infections, with the most pronounced increases occurring during FY 2003 and FY 2004 (Table 2). The overall incidence of health care–associated hospital-onset MRSA infections decreased nonsignificantly (p = 0.47); however, health care–associated community-onset and community-associated MRSA significantly increased (p<0.001), particularly after FY 2003.

The incidence of noninvasive hospital-onset infections did not follow any apparent increasing or decreasing trend (p = 0.08; Table 2). However, driven by noninvasive MRSA infections, the overall incidence of noninvasive community-onset *S. aureus* infections increased significantly (p<0.001). Incidence after FY 2003 rapidly increased from 218 to 546, peaking at 644 cases per 100,000 veterans in FY 2005. After FY 2000, incidence of noninvasive community-onset MRSA infections increased 4-fold from 100 to 397 cases per 100,000 veterans. Incidence of noninvasive community-onset MSSA infections did not change (p = 0.83; Table 2).

A total of 1,703 (61%) noninvasive infections were classified as SSTIs of which 1,112 (65%) were caused by MRSA. Changes in incidence of overall noninvasive MRSA infections were driven by increases in MRSA SSTIs (Figure 2). Incidence per 100,000 veterans significantly increased from 90 cases in FY 1999 to 345 in FY 2008 (p<0.0001); the largest increase began in FY 2003 and incidence peaked at 440 cases in FY 2005.

**Figure 2:** Incidence per 100,000 veterans of skin and soft tissue infections (SSTIs) cause by methicillin-resistant *Staphylococcus aureus* (MRSA) and methicillin-susceptible *S. aureus* (MSSA), Veterans Affairs Maryland Health Care System, fiscal years 1999–2008.

## Discussion

We have described the incidence of all *S. aureus* infections during a 10-year period in a large US-based population (49,000 persons) using person-level data including clinical culture and administrative data to identify and classify infections. Our results suggest significant increases in overall *S. aureus* infections from FY 1999 through FY 2008; the largest increases were associated with community-onset MRSA infections of skin and soft tissue and an overall decrease in incidence of invasive *S. aureus* infections.

During the study period, we implemented many new hospital infection–control practices—including the use of alcohol-based hand gels for hand hygiene (2003), central line bundles (2006), MRSA surveillance cultures (intensive care units in 2003, expanded to acute care in 2007), and chlorhexidine bathing of all surgical patients (2009)—which may have contributed to the decreased incidence of invasive infections. Previous studies suggest that improved infection control practices have contributed to fewer catheter-related and central line–associated bloodstream infections (*20**,**21*). However, attributing the decrease to a single practice is difficult, if not impossible, and few published studies exist with which we can compare our invasive *S. aureus* results.

Laupland et al. estimated an annual incidence of invasive *S. aureus* of 28 cases per 100,000 population, but their results may not be comparable because they were based primarily on MSSA infections (*12*). Klevens et al. estimated an incidence of invasive MRSA infection of 32 cases per 100,000 population, 75% were bacteremias, and 27% were hospital-acquired on the basis of 2005 data from 9 US cities (*13*). Their study reported a noticeably higher incidence of invasive MRSA in Baltimore, Maryland, USA, of 117 per 100,000 compared with estimates of 20–50 per 100,000 population at other sites. Our estimated rate of invasive MRSA infection (160 cases/100,000 veterans) is commensurate during the same year (2005), and our calculated proportion of invasive hospital-onset MRSA in 2005 (20%) also was similar. Klevens et al. reported estimates per 100,000 persons; therefore a direct comparison of rates of invasive hospital-onset MRSA infection is not feasible. However, our rates are potentially higher given the inpatient days adjustment accounting for the first 48 hours in the definition for hospital-onset infection. Another study by Laupland et al. reported an annual incidence of *S. aureus* bloodstream infections during 2000–2006 of 20 per 100,000 population (*14*). However, few MRSA cases were identified in this study, thereby making it difficult to compare with our results for which MRSA caused almost half of infections.

For several reasons, our incidence rates of invasive infections are higher than those previously reported. Black race has previously been reported as a marker for increased risk for invasive MRSA infections (*13**,**22*). Therefore, we would expect a higher incidence of all *S. aureus* infections, given that in our study 49% of patients were black. Additionally, given that our study was performed on data from a primarily urban population located in or around Baltimore, we could attribute the higher incidence of infections to suspected risk factors that are more prevalent in this location, including intravenous drug use.

Our study identified increases in noninvasive *S. aureus* infections, particularly around 2003, which most likely are associated with the emergence of the USA300 MRSA clone that has led to increases in community-associated MRSA, specifically in SSTIs (*1**,**22*). Also, despite dramatic increases in noninvasive community-onset MRSA infections, we did not observe a proportionate increase in invasive community-onset MRSA as might be expected if USA300 MRSA had the same propensity as non-USA300 MRSA to invade the bloodstream. No population-based studies have been published with which to compare overall noninvasive *S. aureus* infections, and few exist for comparison of noninvasive MRSA infections. For instance, Liu et al. recently reported annual (2004–2005) incidence rates of community-acquired and hospital-acquired MRSA among residents in San Francisco, California, USA, community-associated of 316 and 31 cases per 100,000 population, respectively, for which most cultures were from skin and soft tissue (*8*). The results of our study for community-onset MRSA are slightly lower but similar. Crum et al. reported a dramatic increase during 2002–2004 in community-associated MRSA infections, of which most were classified as SSTIs, and an incidence rate of 155 cases per 100,000 persons from 2004 data (*7*). The FY 2004 incidence of noninvasive community-associated MRSA in our study was 188 cases per 100,000 veterans, which is similar to that reported by Crum et al.

Our study adds new information to the existing literature and has several strengths. A major strength is its calculation of annual incidence of all *S. aureus* infections for a 10-year period by using actual numbers of patients at risk, admissions, and inpatient days. Our estimates of incidence are more accurate than those in previous studies, which were based on census-level data (*11**–**13**,**23*). Noninvasive infections were identified by using an automated approach that required both positive clinical culture and confirmed ICD-9-CM code for infection. This definition is more rigorous, thereby producing higher positive predictive values than clinical cultures alone, particularly for infections of bone and skin or soft tissue (*16*). In addition, this automated approach enabled us to identify and classify types of *S. aureus* infection, which is useful for understanding the overall population distribution of infection. Access to comprehensive, patient-level information, including prior hospitalizations, prior MRSA infections, and surgeries, allowed us determine the epidemiologic class for all MRSA infections. This study was performed in a population receiving standardized health care; therefore, findings should be free of bias associated with access to care or duration and type of treatment received.

Our study also has several limitations. First, the VAMHCS population of adult, mostly male patients living in the mid-Atlantic region does not fully represent the overall US population. We are unable to extrapolate these findings to children, a population for which an increase of community-onset MRSA SSTIs has been reported (*24*). Previous reports suggest that men are at higher risk than women for *S. aureus* infections; therefore, our estimates may overestimate true rates for women (*8**,**12**,**13*). Second, although we did not perform molecular typing on the *S. aureus*–positive isolates, we expect that a significant proportion (>80%) of noninvasive MRSA infections were caused by the USA300 MRSA strain (*1*). Given that USA300 MRSA reportedly varies across the United States, our findings may not be generalizable to populations in which MRSA strains differ. Third, we may have underestimated incidence because our definition of *S. aureus* infection required a positive clinical or blood culture. However, given the standardized access to care in the VAMHCS, we expect that cultures were uniformly collected in patients who had clinical signs or symptoms of infection. Fourth, the clinical culturing rate may have increased during our study period, which would contribute to overestimates in the incidence of *S. aureus* infection, particularly noninvasive infections. Johnston et al. observed an increase in the absolute number of SSTI cultures obtained in the VAMHCS Emergency Care Service (*1*). However, they determined that the proportion of MRSA infections increased, even though the proportion of MSSA remained the same, which suggests a true increase in MRSA. We observed similar patterns: whereas MRSA infections increased, MSSA infections remained relatively stable, and the annual number of *S. aureus* cultures did not significantly change.

In conclusion, this large, population-based study demonstrated an increase in the overall incidence of *S. aureus* infections during FY 1999–FY 2008, which was driven by a rapid increase in noninvasive, community-onset, MRSA skin and soft tissue infections. This increase was most striking during and after 2003, which is coincident with the time during which the USA300 clone became a major contributor to noninvasive *S. aureus* infections. Despite this increase, incidence of invasive community-onset MRSA infections did not significantly increase, and the overall MSSA infections and noninvasive MSSA infections remained generally stable. These results suggest a shift in the distribution of *S. aureus* infections to more noninvasive community-onset MRSA infections. This information is useful for interpreting changes in the epidemiology of *S. aureus* infections, which may help guide additional prevention strategies focused on reducing community-onset *S. aureus* infections. To further understand these trends, additional studies are warranted to identify risk factors for *S. aureus* infection and to describe the epidemiology of *S. aureus* infections across the entire population.

## References

[1] Johnson JK, Khoie T, Shurland S, Kreisel K, Stine OC, Roghmann MC. Skin and soft tissue infections caused by methicillin-resistant *Staphylococcus aureus* USA300 clone. Emerg Infect Dis. 2007;13:1195–200.1795309110.3201/eid1308.061575PMC2828080

[2] McCaig LF, McDonald LC, Mandal S, Jernigan DB. *Staphylococcus aureus*–associated skin and soft tissue infections in ambulatory care. Emerg Infect Dis. 2006;12:1715–23.1728362210.3201/eid1211.060190PMC3372331

[3] Jarvis WR, Schlosser J, Chinn RY, Tweeten S, Jackson M. National prevalence of methicillin-resistant *Staphylococcus aureus* in inpatients at US health care facilities, 2006. Am J Infect Control. 2007;35:631–7. 10.1016/j.ajic.2007.10.00918063126

[4] Klevens RM, Edwards JR, Tenover FC, McDonald LC, Horan T, Gaynes R. Changes in the epidemiology of methicillin-resistant *Staphylococcus aureus* in intensive care units in US hospitals, 1992–2003. Clin Infect Dis. 2006;42:389–91. 10.1086/49936716392087

[5] Kuehnert MJ, Hill HA, Kupronis BA, Tokars JI, Solomon SL, Jernigan DB. Methicillin-resistant-*Staphylococcus aureus* hospitalizations, United States. Emerg Infect Dis. 2005;11:868–72.1596328110.3201/eid1106.040831PMC3367609

[6] Noskin GA, Rubin RJ, Schentag JJ, Kluytmans J, Hedblom EC, Smulders M, The burden of *Staphylococcus aureus* infections on hospitals in the United States: an analysis of the 2000 and 2001 Nationwide Inpatient Sample Database. Arch Intern Med. 2005;165:1756–61. 10.1001/archinte.165.15.175616087824

[7] Crum NF, Lee RU, Thornton SA, Stine OC, Wallace MR, Barrozo C, Fifteen-year study of the changing epidemiology of methicillin-resistant *Staphylococcus aureus.* Am J Med. 2006;119:943–51. 10.1016/j.amjmed.2006.01.00417071162

[8] Liu C, Graber CJ, Karr M, Diep BA, Basuino L, Schwartz BS, A population-based study of the incidence and molecular epidemiology of methicillin-resistant *Staphylococcus aureus* disease in San Francisco, 2004–2005. Clin Infect Dis. 2008;46:1637–46. 10.1086/58789318433335

[9] Richards MJ, Edwards JR, Culver DH, Gaynes RP. Nosocomial infections in medical intensive care units in the United States. National Nosocomial Infections Surveillance System. Crit Care Med. 1999;27:887–92. 10.1097/00003246-199905000-0002010362409

[10] Jacobsson G, Dashti S, Wahlberg T, Andersson R. The epidemiology of and risk factors for invasive *Staphylococcus aureus* infections in western Sweden. Scand J Infect Dis. 2007;39:6–13. 10.1080/0036554060081002617366006

[11] Laupland KB, Zygun DA, Davies HD, Church DL, Louie TJ, Doig CJ. Population-based assessment of intensive care unit–acquired bloodstream infections in adults: incidence, risk factors, and associated mortality rate. Crit Care Med. 2002;30:2462–7. 10.1097/00003246-200211000-0001012441755

[12] Laupland KB, Church DL, Mucenski M, Sutherland LR, Davies HD. Population-based study of the epidemiology of and the risk factors for invasive *Staphylococcus aureus* infections. J Infect Dis. 2003;187:1452–9. 10.1086/37462112717627

[13] Klevens RM, Morrison MA, Nadle J, Petit S, Gershman K, Ray S, Invasive methicillin-resistant *Staphylococcus aureus* infections in the United States. JAMA. 2007;298:1763–71. 10.1001/jama.298.15.176317940231

[14] Laupland KB, Ross T, Gregson DB. *Staphylococcus aureus* bloodstream infections: risk factors, outcomes, and the influence of methicillin resistance in Calgary, Canada, 2000–2006. J Infect Dis. 2008;198:336–43. 10.1086/58971718522502

[15] Morin CA, Hadler JL. Population-based incidence and characteristics of community-onset *Staphylococcus aureus* infections with bacteremia in 4 metropolitan Connecticut areas, 1998. J Infect Dis. 2001;184:1029–34. 10.1086/32345911574918

[16] Tracy LA, Furuno JP, Harris AD, Singer M, Langenberg P, Roghmann MC. Predictive ability of positive clinical culture results and International Classification of Diseases, Ninth Revision, to identify and classify noninvasive *Staphylococcus aureus* infections: a validation study. Infect Control Hosp Epidemiol. 2010;31:694–700. 10.1086/65320620476891PMC3010845

[17] Nelder JA, Wedderburn RW. Generalized linear models. J Roy Statist Soc A. 1972;135:370–84. 10.2307/2344614

[18] Chatfield C. The analysis of time series: an introduction. 6th ed. Boca Raton (FL): Chapman and Hall/CRC; 2003.

[19] Kedem B, Fokianos K. Regression models for time series analysis. New York: John Wiley & Sons; 2002.

[20] Burton DC, Edwards JR, Horan TC, Jernigan JA, Fridkin SK. Methicillin-resistant *Staphylococcus aureus* central line–associated bloodstream infections in US intensive care units, 1997–2007. JAMA. 2009;301:727–36. 10.1001/jama.2009.15319224749

[21] Pronovost P. Interventions to decrease catheter-related bloodstream infections in the ICU: the Keystone Intensive Care Unit Project. Am J Infect Control. 2008;36:S171–5. 10.1016/j.ajic.2008.10.00819084146

[22] King MD, Humphrey BJ, Wang YF, Kourbatova EV, Ray SM, Blumberg HM. Emergence of community-acquired methicillin-resistant *Staphylococcus aureus* USA 300 clone as the predominant cause of skin and soft-tissue infections. Ann Intern Med. 2006;144:309–17.1652047110.7326/0003-4819-144-5-200603070-00005

[23] Klein E, Smith DL, Laxminarayan R. Hospitalizations and deaths caused by methicillin-resistant *Staphylococcus aureus*, United States, 1999–2005. Emerg Infect Dis. 2007;13:1840–6.1825803310.3201/eid1312.070629PMC2876761

[24] Herold BC, Immergluck LC, Maranan MC, Lauderdale DS, Gaskin RE, Boyle-Vavra S, Community-acquired methicillin-resistant *Staphylococcus aureus* in children with no identified predisposing risk. JAMA. 1998;279:593–8. 10.1001/jama.279.8.5939486753

# Research article: *Staphylococcus aureus* Infections in US Veterans, Maryland, USA, 1999–20081

**DOI:** 10.3201/eid1703.100502

**Authors:** LaRee A. Tracy, Jon P. Furuno, Anthony D. Harris, Mary Singer, Patricia Langenberg, Mary-Claire Roghmann

**Affiliations:** Author affiliations: University of Maryland, Baltimore, Maryland, USA (L.A. Tracy, J.P. Furuno, A.D. Harris, P. Langenberg, M.-C. Roghmann);; Veterans Administration Maryland Health Care System, Baltimore (M.-C. Roghmann);; US Food and Drug Administration, Silver Spring, Maryland, USA (M. Singer)

**Keywords:** *Suggested citation for this article:* Tracy LA, Furuno JP, Harris AD, Singer M, Langenberg P, Roghmann M-C. *Staphylococcus aureus* infections in US veterans, Maryland, USA, 1999–2008. Emerg Infect Dis [serial on the Internet]. 2011 Mar [*date cited*]. http://dx.doi.org/10.3201/eid1703.100502

## Earning CME Credit

To obtain credit, you should first read the journal article. After reading the article, you should be able to answer the following, related, multiple-choice questions. To complete the questions and earn continuing medical education (CME) credit, please go to www.medscape.org/journal/eid. Credit cannot be obtained for tests completed on paper, although you may use the worksheet below to keep a record of your answers. You must be a registered user on Medscape.com. If you are not registered on Medscape.com, please click on the New Users: Free Registration link on the left hand side of the website to register. Only one answer is correct for each question. Once you successfully answer all post-test questions you will be able to view and/or print your certificate. For questions regarding the content of this activity, contact the accredited provider, CME@medscape.net. For technical assistance, contact CME@webmd.net. American Medical Association’s Physician’s Recognition Award (AMA PRA) credits are accepted in the US as evidence of participation in CME activities. For further information on this award, please refer to http://www.ama-assn.org/ama/pub/category/2922.html. The AMA has determined that physicians not licensed in the US who participate in this CME activity are eligible for *AMA PRA Category 1 Credits*™. Through agreements that the AMA has made with agencies in some countries, AMA PRA credit is acceptable as evidence of participation in CME activities. If you are not licensed in the US and want to obtain an AMA PRA CME credit, please complete the questions online, print the certificate and present it to your national medical association.

## Article Title: *Staphylococcus aureus* Infections in US Veterans, Maryland, USA, 1999–2008

## CME Questions

**1. On the basis of the current retrospective cohort study by Dr. Tracy and colleagues, which of the following statements about the overall incidence of *Staphylococcus aureus* infections in the Veterans Affairs Maryland Healthcare System between fiscal years 1999 and 2008 is most likely correct?**A. Incidence of all *S. aureus* infections significantly decreasedB. Changes in incidence of all *S. aureus* infections were most striking during and following 2005C. 10-year average overall incidence per 100,000 veterans was 749 ± 132D. The findings are applicable to pediatric units**2. You are an infection-control officer in a Maryland veterans hospital. On the basis of the study by Dr. Tracy and colleagues, which of the following statements about trends in invasive vs noninvasive *S. aureus* infections, methicillin susceptibility, and location of onset and infection site is most likely to apply to infection control at your hospital?**A. About half of* S. aureus* infections are likely to be invasiveB. A projected increase in overall *S. aureus* infections is most likely to be driven by invasive, methicillin-sensitive, hospital-onset infectionsC. More than half of all noninvasive infections are likely to be skin and soft tissue infectionsD. Hospital-onset invasive MRSA infections are likely to significantly increase**3. As the infection-control officer described in question 2, which of the following hospital infection-control practices do you think would be most likely to reduce incidence of invasive *S. aureus* infections, as based on their use in the VA hospital studied by Dr. Tracy and colleagues?**A. Hand-washing with soap and water rather than using alcohol-based hand gelsB. Central-line bundlesC. Surveillance cultures for methicillin-sensitive *S. aureus*D. Soap-and-water rather than chlorhexidine bathing for surgical patients

## Activity Evaluation

**Table:** 

1. The activity supported the learning objectives.				
Strongly Disagree				Strongly Agree
1	2	3	4	5
2. The material was organized clearly for learning to occur.				
Strongly Disagree				Strongly Agree
1	2	3	4	5
3. The content learned from this activity will impact my practice.				
Strongly Disagree				Strongly Agree
1	2	3	4	5
4. The activity was presented objectively and free of commercial bias.				
Strongly Disagree				Strongly Agree
1	2	3	4	5
